# Mayer-Rokitansky-Kuster-Hauser syndrome type II with crossed fused renal ectopia: A rare case report

**DOI:** 10.1016/j.radcr.2023.01.107

**Published:** 2023-03-07

**Authors:** Hari Soekersi, Radi Trihadrian

**Affiliations:** Department of Radiology, Faculty of Medicine Universitas Padjadjaran, Hasan Sadikin General Hospital, Jl. Pasteur No. 38, Pasteur, Kec. Sukajadi, Kota Bandung, Bandung, 40161, Indonesia

**Keywords:** Crossed Fused Renal Ectopia, Mayer-Rokitansky-Küster-Hauser syndrome, Magnetic Resonance Imaging

## Abstract

Mayer-Rokitansky-Küster-Hauser (MRKH) syndrome is a congenital disorder characterized by agenesis or aplasia of the uterus. Patients usually present with primary amenorrhea. MRKH can be isolated (type I) or accompanied by other malformations (type II) involving the kidney, skeletal, and vascular systems. Magnetic resonance imaging (MRI) is the mainstay of imaging modality in evaluating this syndrome. A 20-year-old woman presented with cyclic abdominal pain and primary amenorrhea. Secondary sexual characteristics and hormone evaluation were normal. Ultrasound and MRI were conducted and revealed no normal uterus structure with ectopic ovarium and crossed fused ectopic renal.

## Introduction

Mayer-Rokitansky-Küster-Hauser (MRKH) syndrome is a congenital disorder characterized by agenesis or aplasia of the uterus and upper part of the vagina in females with a normal female karyotype (46,XX). MRKH syndrome is a very rare disease and the prevalence is generally considered to be in 1 in 5000 live female births but it remains poorly investigated. The etiology of MRKH syndrome remains unclear. It is hypothesized that anti-Müllerian hormone (AMH) inhibits the development of Müllerian structures, which led to the idea of overexpression of AMH and its receptors; however, studies have failed to find evidence for this hypothesis [1].

Patients with MRKH syndrome usually present with primary amenorrhea. MRKH syndrome can be isolated (type I) or accompanied by other malformations (type II), involving the kidney (renal ectopic or agenesis), skeletal and vascular system [2]. MRI is currently the mainstay of imaging in evaluating this syndrome, as it is free of radiation, noninvasive and has multiplanar capabilities [3]. In this case report, we present a woman with MRKH syndrome type II, ectopic ovarium, and crossed fused renal ectopia, as well as the modalities used to confirm the diagnosis. To the best of our knowledge, this is a rare form of MRKH syndrome and only few cases have been reported in the literature.

## Case

A 20 years old woman presented to our institution with cyclic abdominal pain since one year ago. The pain was not associated with menstruation since she never had menstruation (primary amenorrhea). The patient had no previous history of gynecological surgery, radiotherapy, or chemotherapy. Physical examination revealed no abnormality with symmetrical tanner stage IV breast. She had normal distribution of axillary and pubic hair with grossly normal external female genitalia. Bimanual examination was not conducted due to patient rejection. Body weight and height was 46 kg and 163 cm, respectively. Hormonal evaluation was normal and karyotype test showed 46,XX chromosome.

**Fig. 1:**
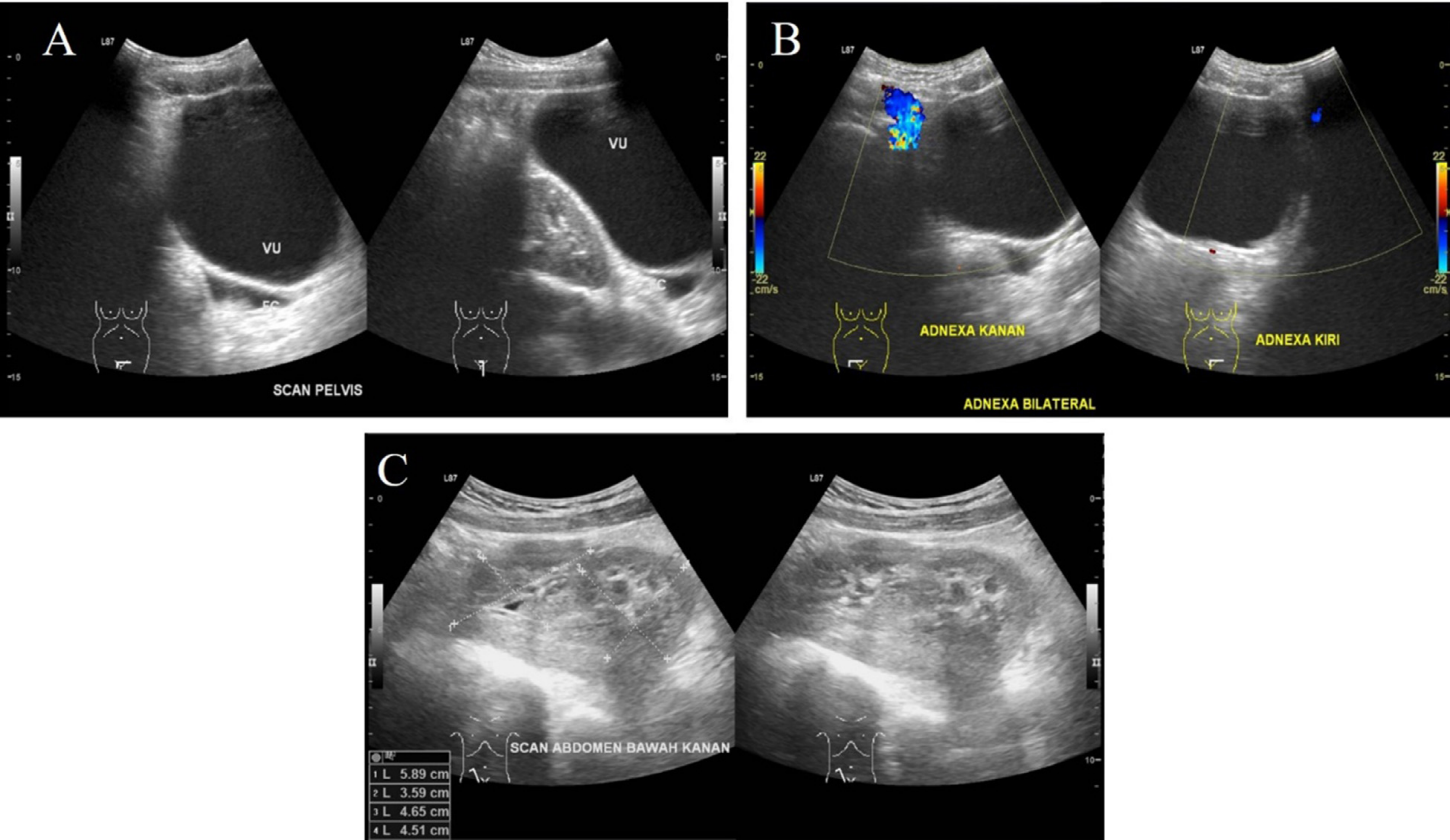
(A and B) Structure of normal uterus was not seen. (C) Lower abdominal ultrasound revealed ectopic kidney within the right pelvic cavity, ureter was difficult to be observed.

Pelvic ultrasound findings indicated uterine agenesis with 2 ectopic kidneys within the right pelvic cavity (Fig. 1), suggesting MRKH syndrome type II (atypical). Ovaries were not found in adnexa regions. Abdominal ultrasound of the liver, gallbladder, pancreas, spleen, and vesica urinary were within normal limit.

**Fig. 2:**
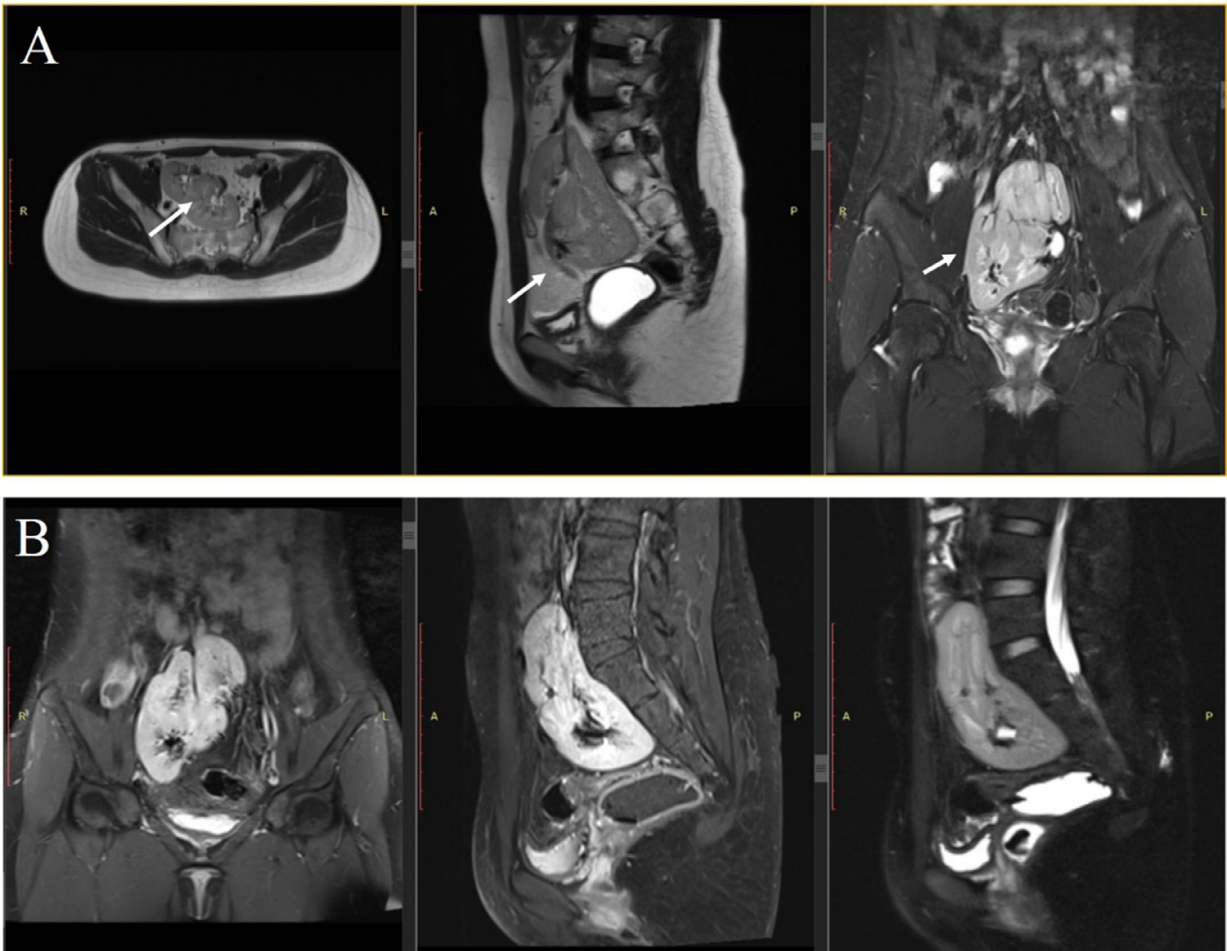
Contrast MRI (A) normal uterus and adnexa were not found on T2FS. Ectopic kidney was seen within the right pelvic cavity (white arrow). (B) Similar finding was found on T1W1.

**Fig. 3:**
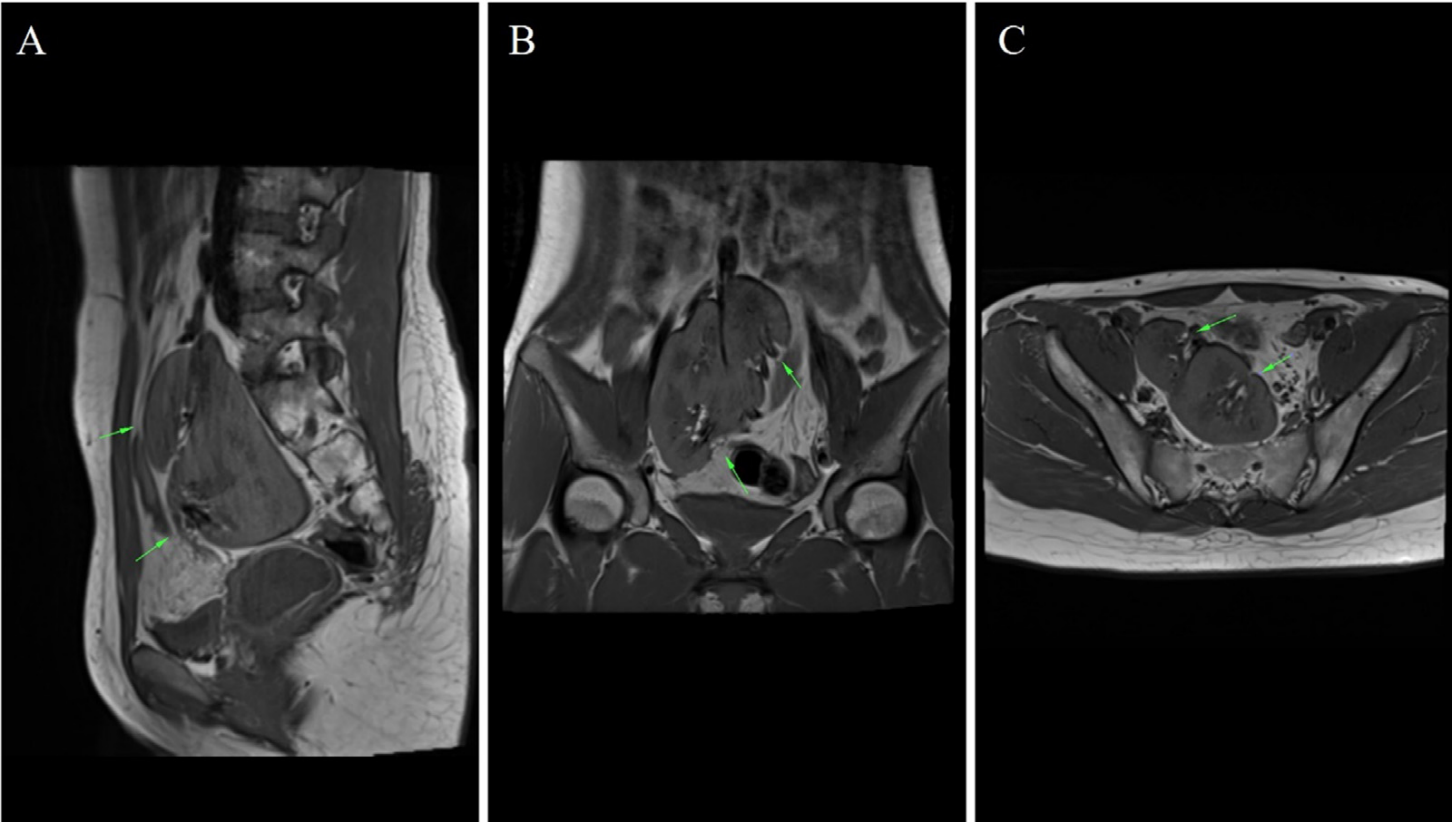
MRI showed cross-fused ectopic kidney within the pelvic cavity on sagittal (A), coronal (B), and transverse (C) section. Two renal pelvis was found (green arrow).

MRI with and without contrast was conducted for further confirmation of MRKH and to identify other associated anomalies. MRI revealed no normal structures of uterus and vagina. Right and left ovarium were found in right and left iliac fossas, respectively (Fig. 2). MRI also showed 2 fused kidney structures within the pelvic areas, suggesting crossed fused renal ectopia. Based on those findings, the patient was diagnosed with MRKH syndrome type II. (Fig. 3).

## Discussion

MRKH syndrome, or Müllerian aplasia, is a syndrome that affects females with normal karyotype (46,XX). Type I is characterized by uterovaginal aplasia, while Type II is additionally related to extragenital anomalies, most commonly renal (30%-40%), skeletal, ear, and cardiac anomalies [4]. The oviducts, uterus, cervix, and upper two-thirds of the vagina origin from the paramesonephric (Müllerian) ducts (PMD), whereas the lower part of the vagina origins from the urogenital sinus. The caudal part of the 2 PMDs fuses to form the uterus, cervix and upper vagina, whereas the upper parts of the PMDs form the 2 oviducts. MRKH syndrome is caused by either complete agenesis or aplasia of the PMDs to form the uterus and upper vagina. The etiology of MRKH syndrome remains elusive, however increasing reports of familial clustering point towards genetic causes and the use of various genomic techniques has allowed the identification of promising recurrent genetic abnormalities in some patients.

Patients with MRKH syndrome typically present during adolescence with primary amenorrhea and normal secondary sexual characteristics. Other complaints at referral include dyspareunia/apareunia and (cyclic) abdominal pain. Finally, patients (typically younger children) may be referred after an incidental finding of vaginal or uterus agenesis. Median age at referral has been reported to be 17.5 years (interquartile range: 16-19) [1]. Study by Khan et al, reported mean age of presentation of 22.2 years, while in this case patient presented at 20 years of age with primary amenorrhea and cyclic abdominal pain.

Transperineal or transabdominal ultrasonography (US) is performed revealing the absence of the uterus and presence of ovaries. Magnetic resonance imaging (MRI) of the internal genitalia is considered the golden standard method for the diagnosis of uterovaginal agenesis in MRKH syndrome. MRI also shows the ovaries and extragenital malformations, and has high interrater agreement with laparoscopy. Uterus agenesis/aplasia in MRKH syndrome has 2 phenotypic presentations. Two aplastic uterine buds on the pelvic sidewall derived from the Müllerian ducts (often seen in type I) or complete absence of one or both Müllerian ducts (often seen in type II associated with ipsilateral kidney malformations). Presence of uterine remnants have been reported in 48%-95% of the patients and is associated with a risk of cyclic (catamenial) abdominal pain due to the presence of active endometrium [1]. Detail evaluations of uterine remnants should be done for presence, site, volumes and differentiations into layers (myometrium, functional zone, and endometrium). On TIW images, the uterine remnants may appear as solid elongated to ovoid structure with isointense to low signal intensities. T2W images showed the potential cavitation within the uterine buds. The cavitation appears as a target pattern consisting of a central area of T2W hyperintense signal intensity representing the endometrium surrounded by intermediate signal of junctional zone and medium to high signal intensity of muscular layer. Presence of connecting/converging fibrous bands between the uterine remnants should also be looked for [5]. While in this, case uterine remnant was not found despite symptom of cyclic abdominal pain.

Endometriosis is another feature which may occur in MRKH syndrome, especially in patients with uterine remnants and active endometrium [1]. However, in this case, remnant of the uterus or endometriosis was not found on MRI despite the presence of cyclic abdominal pain. In MRKH syndrome, both ovaries are typically present and well-functioning. However, their anatomical position is usually more cranial than the normal position and they are often found lateral, rather than medial, to the external iliac arteries, probably due to the lack of Fallopian tube development. Ovary anomalies are rare and only found in ∼ 5%-10%. In this case no abnormality was found within both ovaries. Follicular cyst indicated a normal functioning ovarium in this patient. Study by Hall-Craggs et al reported that 2 patients (33%) had no vagina or only a vaginal dimple. The other 42 patients had a vagina seen at MR imaging, and the mean vaginal length was 2.0 cm (range, 1.0-6.5 cm) [6]. While in this case, patient had normal appearing vagina with normal length without the presence of cervix.

The difference between MRKH type I and type II is the presence of extragenital anomaly. In MRKH type II, there is incomplete aplasia associated with other malformations including renal (unilateral agenesis, ectopia of the kidneys or horseshoe kidney), skeletal (Klippel–Feil anomaly or fused vertebrae, mainly cervical; scoliosis), hearing defects, cardiac and digital anomalies (syndactyly and polydactyly). It is referred to as MURCS association or genital renal ear syndrome. Primary damage to the mesoderm (paraxial, intermediate, and lateral) is held responsible for skeletal, pulmonary, renal and Müllerian dysplasias present in MRKH syndrome. Oppelt et al reported that malformations of the renal system were the most frequent type of accompanying malformation, seen in 19 patients (40%-60%), followed by 19 different skeletal changes in 15 patients [7]. Simultaneous pulmonary anamolies, renal agenesis or dysplasia, and MRKH syndrome has been reported very rarely in literature. The common types of renal anomalies may include renal agenesis and ectopic pelvic kidney.

In this case, crossed fused ectopic kidney was found on MRI. This is a very rare finding in MRKH syndrome type II. Finding in this case may give additional insight to the possible associated renal anomaly in MRKH syndrome type II without urologic symptom. Mudoni et al reported a case of abdominal tomography scan with contrast medium that showed the presence of renal ectopia with kidneys having been fused with each other, both being located on the right without other associated anomalies. Study by Agwany et al, reported similar finding in MRKH syndrome type II without significant symptom in the patient [8]. Another study by Liang et al, reported a magnetic resonance urography that showed right to left renal crossed-ectopia with inferior fusion, and hydronephrosis and hydroureter from the superior kidney with Grade IV vesicoureteral reflux [9]. Case report by Muhammad et al demonstrated a case of extensively fused ectopic renal parenchyme without intervening septum in between. The collecting systems of the kidneys united to form a common ureter which followed a short course on the left side to join the urinary bladder at left vesicoureteric junction. No ureter was outlined on the right side [10]. Thus, cross fused renal ectopia is rare and when it present, the presentation and finding varies among patients.

## Conclusion

This case represents a MRKH syndrome type II patient with ectopic bilateral kidney. The unique presentation in this case was the crossed fused renal ectopia and the absence of mullerian remnant or endometriosis despite the presence of cyclic abdominal pain. This indicated that other anomaly could be responsible for patient cyclic pain or the MRI in this case failed to identify mullerian remnant. Further investigation such as diagnostic laparotomy may be needed to confirm this assumption. This report highlights the possibility of crossed fused renal ectopia occurence in MRKH syndrome and the importance of MRI in confirming the diagnosis.

## Patient consent

The author confirm that written informed consent for publication of this case report has been obtained from the patient.
